# Reduction patterns of Japanese encephalitis incidence following vaccine introduction into long-term expanded program on immunization in Yunnan Province, China

**DOI:** 10.1186/s40249-019-0608-7

**Published:** 2019-12-10

**Authors:** Xiao-Ting Hu, Qiong-Fen Li, Chao Ma, Zhi-Xian Zhao, Li-Fang He, Ting-Ting Tang, Wen Yu, Philip Owiti

**Affiliations:** 1 Expanded Program on Immunization, Yunnan Center for Disease Control and Prevention, NO.158 DongSi Street, XiShan District, Kunming City, Yunnan Province China; 2 National Immunization Program, Chinese Center for Disease Control and Prevention, 27 Nanwei Road, Xicheng District, Beijing, China; 3 The International Union Against Tuberculosis and Lung Disease, Paris, France; 4 The National Tuberculosis, Leprosy and Lung Disease Program, Nairobi, Kenya

**Keywords:** Vaccination program, Incidence, Surveillance system, Epidemiology, Operational research

## Abstract

**Background:**

Japanese encephalitis (JE) is a leading cause of childhood viral encephalitis both at global level and in China. Vaccination is recommended as a key strategy to control JE. In China most JE cases have been reported in southwest provinces, which include Yunnan. In this study, we quantify the epidemiological shift of JE in Yunnan Province from 2005 to 2017, covering before and after the introduction of JE vaccination into routine Expanded Program on Immunization (EPI) in 2007.

**Methods:**

We used routinely collected data in the case-based JE surveillance system from 2005 through 2017 in Yunnan. Cases were reported from hospital and county-level Centers for Disease Control in line with the National JE Surveillance Guideline. Epidemiological data were extracted, analysed and presented in appropriate ways. Immunization coverage was estimated from actual JE doses administered and new births for each year.

**Results:**

A total 4780 JE cases (3077 laboratory-confirmed, 1266 clinical and 437 suspected) were reported in the study period. Incidence of JE (per 100 000 population) increased from 0.95 in 2005 to 1.69 in 2007. With increase in vaccination coverage, incidence rates decreased steadily from 1.16 in 2009 to 0.17 in 2017. However, seasonality remained similar across the years, peaking in June–September. Banna (bordering Myanmar and Laos), Dehong (bordering Myanmar), and Zhaotong (an inland prefecture) had the highest incidence rates of 2.3, 1.9, and 1.6, respectively. 97% of all cases were among local residents. As vaccination coverage increased (and incidence decreased), proportion of JE cases among children < 10 years old decreased from 70% in 2005 to 32% in 2017, while that among adults ≥20 years old increased from 12 to 48%. There were a large number of JE cases with unknown treatment outcomes, especially in the earlier years of the surveillance system.

**Conclusions:**

The 13-year JE surveillance data in Yunnan Province showed dramatic decrease of total incidence and a shift from children to adults. Improving vaccination coverage, including access to adults at risk, and strengthening the JE surveillance system is needed to further control or eliminate JE in the province.

## Multilingual abstracts

Please see Additional file 1 for translations of the abstract into the five official working languages of the United Nations.

## Background

Japanese encephalitis (JE) is a leading cause of childhood viral encephalitis. It is caused by a flavivirus spread to humans by infected *Culex* mosquitoes. It is related to the viruses causing dengue, yellow fever, and West Nile fevers [1]. The first case of JE was documented in 1871 in Japan and although it is rarely symptomatic, the case-fatality rate can be as high as 30% [2]. Permanent neurologic or psychiatric sequelae can also occur in 30–50% of those with the encephalitis. There is no cure currently for the disease, and treatment is aimed at relieving severe clinical signs and supporting the patient to overcome the infection [3, 4].

Globally, 75% of cases occur in children and adolescents leading to an annual incidence of 5.4 cases per 100 000 population [4]. There are 24 countries in the World Health Organization Southeast Asia and Western Pacific regions that have endemic JE virus transmission, exposing more than three billion people to risks of infection. In Asia, the virus is the main cause of viral encephalitis resulting into an estimated 68 000 clinical cases every year [3].

In China, the number of reported JE cases ranged between 1625 to 2178 in 2011–2013, with the reported incidence rates increasing from 0.12 to 0.16 per 100 000 population, respectively [5]. In 2011 and 2012, most of these cases were reported in southwest provinces, which include Yunnan Province which accounted for 17% of the cases. In Yunnan Province, a total 47 885 JE cases were reported between 1952 and 2015, 14% of whom died [6].

Safe and effective vaccines have been available to prevent JE, although not routinely used in all affected countries including those bordering China, substantial progress has been made in establishing and strengthening JE immunization programs. Countries which have had major epidemics in the past, but which have controlled the disease primarily by vaccination, include China, Republic of Korea [7], Japan and Thailand. Other countries that still have periodic epidemics include Viet Nam [8], Cambodia, Myanmar, India, Nepal, and Malaysia. Nepal [9, 10], Cambodia and the Laos established national JE immunization programs in 2015–2016 after conducting catch-up campaigns targeting children aged < 15 years. Myanmar, Indonesia, and the Philippines [11] introduced JE vaccination in 2018. China as a country included a two-dose schedule of JE vaccine into routine Expanded Program on Immunization (EPI) in 2007, administered to children at 8 months and 2 years, respectively.

Yunnan is one of the southwest provinces in China most affected by JE, bordering Myanmar, the Laos and Viet Nam. There is cross-border movement in this province with cases of JE reported in both locals and migrants. However, to-date there has been no publications analyzing cases of JE which include the migrant population in Yunnan. We thus set out, using case-based JE surveillance data from 2005 to 2017 in Yunnan Province, to describe the epidemiological and clinical characteristics of the cases before and after the inclusion of JE vaccination into routine EPI program.

## Methods

### Study design

This was a retrospective study utilizing routinely collected case-based JE data.

### Setting

China is divided into 22 provinces, five autonomous regions, four municipalities, and three special administrative regions. Geographically, all provincial divisions can be grouped into six regions, including North China, Northeast China, East China, South Central China, Southwest China and Northwest China. The provinces are divided into prefectures, districts/counties, communities/townships, and neighborhood committees/villages (urban/rural) [12]. Yunnan Province is in southwest of China, bordering the provinces of Guangxi, Guizhou, Sichuan, and the Tibet Autonomous Region, and the countries of Vietnam, the Laos, and Myanmar. The province, with an approximate population of 48 million people [13], has a tropical to subtropical climate and a diverse biota which is coupled with the complex topography of the China-Myanmar-Laos border [14]. The terrain is largely mountainous, especially in the north and west. Average annual rainfall ranges from 600 to 2300 mm, with over half the rain occurring between June and August. The plateau region has moderate temperatures [13]. The western canyon region is hot at the valley bottoms, but there are freezing winds at the mountaintops, providing favorable environments for the breeding of mosquitoes, a known vector of JE virus [15].

### JE vaccine and coverage estimation

Developed in 1968, JE vaccine was largely unaffordable due to costs. The immunization coverage with the vaccine was low and had no significant impact on the JE pandemic. After inclusion into the EPI in 2008, the national JE incidence rate remained at a low level [16]. Due to the animal reservoirs, JE virus cannot be eliminated but disease could potentially be controlled by universal human vaccination in endemic areas [17]. To estimate the immunization coverage, we used the number of the 1st and 2nd doses of JE vaccine (JE1 and JE2) administered through routine immunization in Yunnan Province each year as numerators, and the published data on number of new births reported in the Statistical Year Book for Yunnan Province as denominators.

### JE surveillance system and data resource

Although JE has been a notifiable disease in China since the 1950s, for many years only aggregate data on JE incidence were submitted to the country’s province level. In 2005, China began to implement a National Notifiable Disease Reporting System (NNDRS), that permitted surveillance data to flow from hospitals and county-level Centers for Disease Control (CDC) to the national CDC, through a web-based computerized reporting system [18]. The National JE Surveillance Guideline was then issued in 2006 and recommended that case-based JE surveillance be conducted throughout the country.

In this surveillance, a suspected case of JE was defined as a person of any age during a mosquito activity season living in JE epidemic areas or had been to the JE epidemic areas within 25 days before the onset, with acute onset of fever and a change in mental status (including symptoms such as confusion, disorientation, coma, or inability to talk) and/or new onset of seizures [19]. Other early clinical findings may include an increase in irritability, somnolence or abnormal behavior greater than that seen with usual febrile illness. Laboratory confirmation use JE virus-specific IgM antibody ELISA test in a single sample of cerebrospinal fluid (CSF) or serum. The data of every suspected JE case are entered into the case-based JE surveillance system. These include age, sex, location/ residence, occupation, vaccination status, date of onset, clinical manifestation of the disease, and laboratory results.

Cases were investigated by the epidemiological team within 48 h of reporting using a standard questionnaire. They would then be followed-up for treatment outcome after 6 months. The outcome of the patients was recorded at the time of discharge. Few patients were released from the hospital against medical advice and their condition could not be assessed – these were dropped from the outcome analysis. Outcome was defined as recovered completely, recovered with neurological sequelae, and death [20]. Neurological sequelae included loss of muscle strength, muscle tone, muscle feeling, tendon reflexes and pathological reflex, aphasia, cranial nerve system symptoms (dysphagia, salivation, lisping, vision loss, hearing loss), mental state, and with/without epilepsy [21].

### Data analysis

For this report, the case-based JE surveillance data reported between January 2005 and December 2017 were analyzed. Demographic and clinical characteristics were presented in frequencies and proportion. Trends in incidence rates were presented in linear graphical form. In line with the current national JE surveillance guideline, case numbers were counted by date of onset and incidences – expressed as the number of cases per 100 000 population – and compared with population denominators provided by China’s National Bureau of Statistics. The spatial distribution was presented using MapInfo 15.0 (Pitney Bowes, Connecticut, US).

## Results

### Trends in the JE cases

**Fig. 1:**
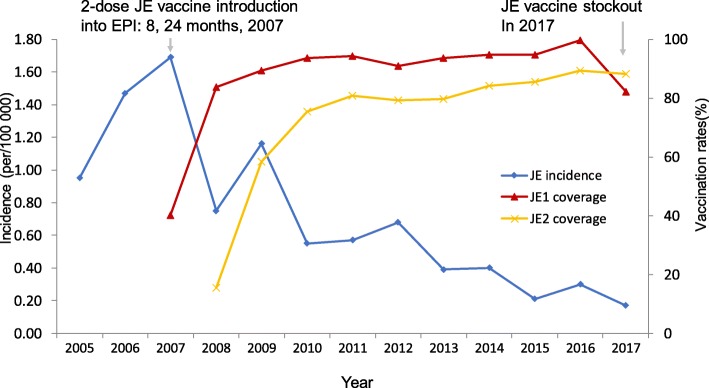
Incidence of Japanese encephalitis and coverage of vaccine before and after introduction of the two-dose vaccination in Expanded Program on Immunization, Yunnan Province. Notes: EPI – Expanded Program on Immunization; JE – Japanese encephalitis; JE1 – 1st dose JE vaccination; JE2 – 2nd dose JE vaccination

**Fig. 2:**
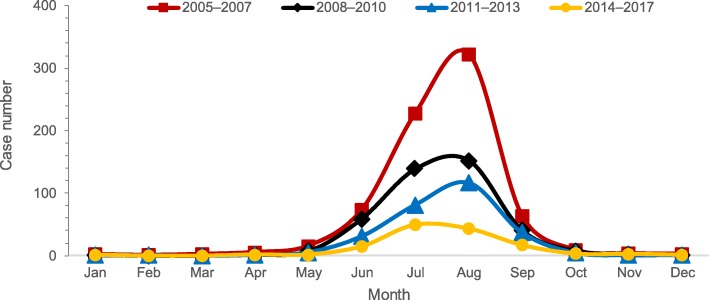
Monthly distribution of Japanese encephalitis cases in Yunnan Province, 2005–2017

In Yunnan Province, a total of 4780 JE cases were reported during 2005–2017. Of these, 3077 (64%) were laboratory-confirmed while 2899 (61%) were males (Table 1). Before the two-dose JE vaccination was introduced as part of routine EPI program in Yunnan in 2007, the reported incidence of JE (per 100 000 population) increased from 0.95 in 2005 to 1.69 in 2007. With the increase in uptake and coverage of vaccination, the incidence rate of JE decreased steadily from 1.16 in 2009 to 0.17 in 2017 (Fig. 1). Even though the seasonality remained similar across the years, peaking in the months of June through September, the number of cases decreased significantly after introduction of JE vaccine (Fig. 2).

**Table 1:** Epidemiological and clinical characteristics of Japanese Encephalitis cases in Yunnan Province, China, 2005–2017

Characteristics	*n*	%
Total number of cases	4780	100.0
Age group, *years*		
0–4	1340	28.0
5–9	1441	30.1
10–14	658	13.8
15–19	288	6.0
≥ 20	1053	22.0
Gender		
Male	2899	60.6
Female	1881	39.4
Nationality of patients		
China	4641	97.1
Myanmar	113	2.4
Laos	25	0.5
others	1	0
Occupation of patients		
Farmer	920	19.2
Student	1585	33.2
Preschool children	1770	37.0
Others	505	10.6
JE Case classification		
Laboratory-confirmed cases	3077	64.4
Clinical cases	1266	26.5
Suspected cases	437	9.1
Travel History 25 Day prior to onset		
Overseas	33	0.7
Another Province	42	0.9
Inside Yunnan Province	217	4.5
No outside travel history	2560	53.6
Not recorded	1928	40.3
Severity of patient classified by doctor		
Unknown	2358	49.3
Mild	749	15.7
Medium	799	16.7
Severe	764	16.0
Very serious	110	2.3
Patient’s JE Vaccination history		
0-dose	1327	27.8
1-dose	215	4.5
2-dose	50	1.0
Unknown	3188	66.7
Outcome		
cured	638	13.3
sequela	1424	29.8
death	326	6.8
Unknown	2392	50.0

### Epidemiological characteristics of the cases

**Fig. 3:**
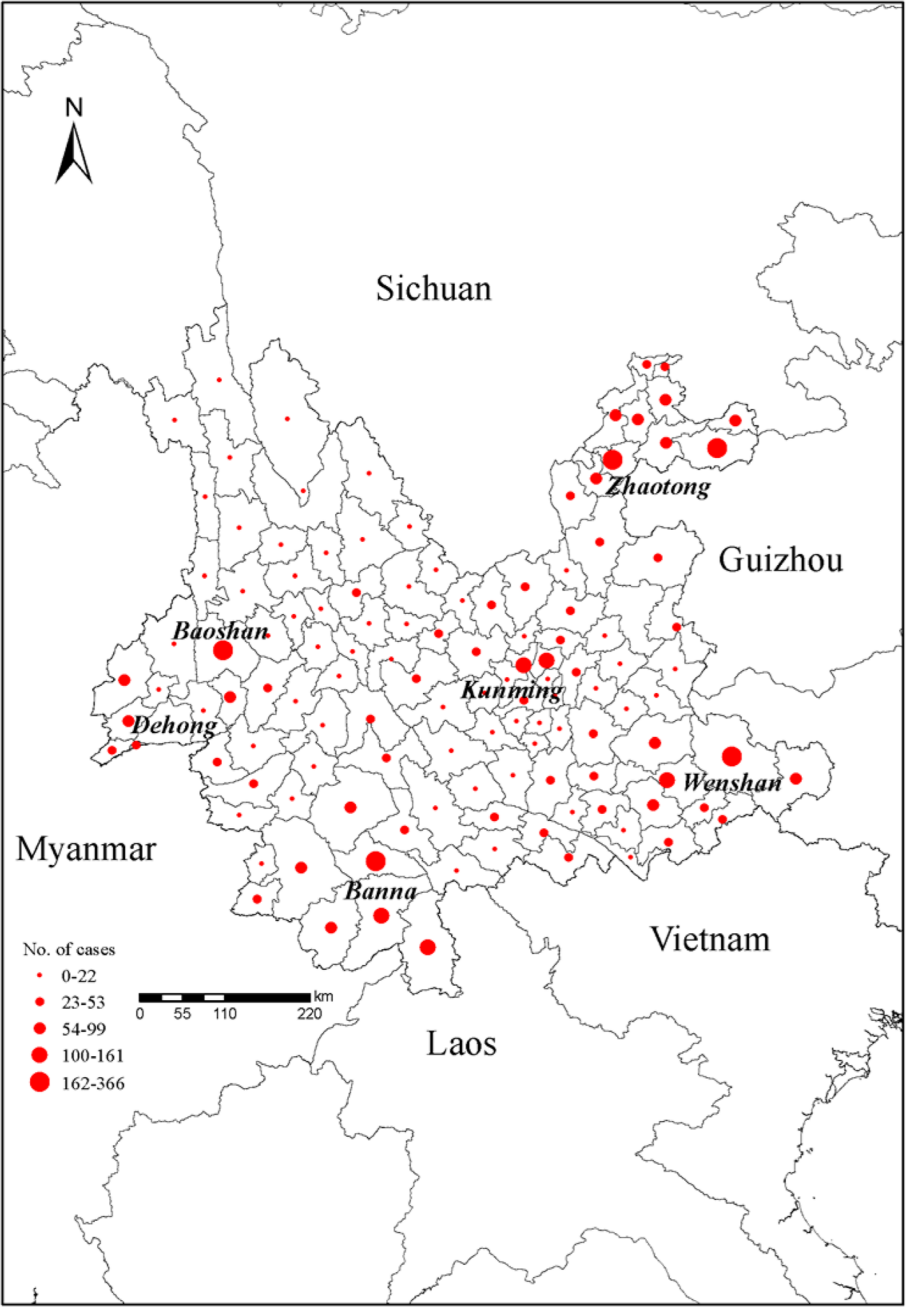
The spatial distribution of Japanese encephalitis cases in Yunnan Province, 2005–2017

JE cases were reported in all the 16 prefectures of Yunnan Province (Fig. 3). Banna (bordering Myanmar and Laos), Dehong (bordering Myanmar), Zhaotong (an inland prefecture) had the highest incidence rates (per 100 000 population) of 2.3, 1.9, and 1.6, respectively. Of all JE cases, 0.9% had travelled outside the province while another 0.7% had travelled overseas in the 25 days prior to onset of JE symptoms.

The majority (97%) of all cases were among the local residents while the rest (139 cases) were from neighboring nationalities (113 cases from Myanmar, 25 from Laos, 1 from the other country).

**Fig. 4:**
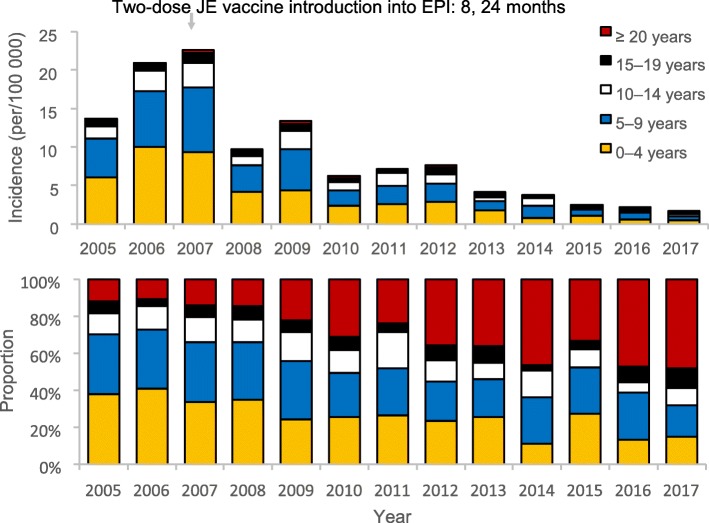
Incidence and proportion of Japanese encephalitis cases by age-group in Yunnan Province, 2005–2017. Notes: EPI – Expanded Program on Immunization

Overall, most cases (58%) affected children < 10 years old while adults ≥20 years old accounted for 22% (Table 1). As the vaccination coverage increased from 40% in 2007 to 82% in 2017 (and incidence of JE decreased), the proportion of JE cases among children < 10 years old decreased from 70% in 2005 to 32% in 2017 (Fig. 4). On the contrary, proportion of cases among adults ≥20 years old increased from 12% in 2005 to 48% in 2017.

Of the 4780 cases, only 4.5% had at least one dose of JE vaccination although for 67% this history was unknown (Table 1). However, among 328 cases who were between 1 and 9 years of age from 2008 to 2017 birth cohorts and thus were eligible for routine JE vaccination, 18% had at least one dose of JE vaccination.

### Treatment outcomes

**Fig. 5:**
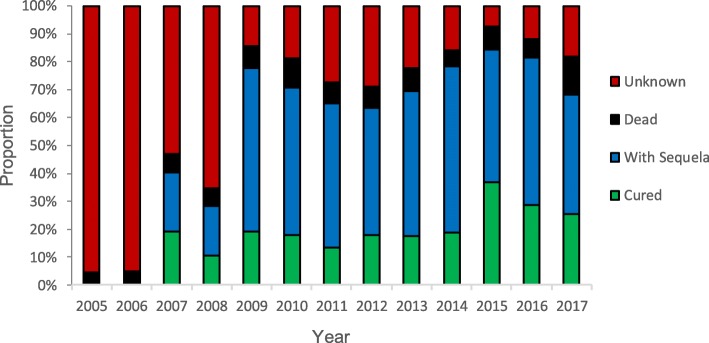
Treatment outcomes of Japanese encephalitis cases in Yunnan Province, 2005–2017

There were a large number of JE cases with unknown treatment outcome, especially in the earlier years of the surveillance system (2005–2006). The proportion cured increased marginally over the years while those with sequela and those who died did not change significantly (Fig. 5). This pattern remained even after eliminating unknown from the denominator (*data not shown*).

## Discussion

Our analysis of JE epidemiology from 2005 to 2017 in Yunnan Province of China showed that, with the increase of JE vaccination coverage, the JE incidence rate decreased steadily. Majority of the cases occurred among Yunnan local residents. The shift of cases to adults, as opposed to children, indicates that the older age-groups are now at more risk. Lastly, the large amount of unknown treatment outcome revealed inherent weaknesses of the JE surveillance system.

This study utilized surveillance data over a long period of time (13 years), giving a good glance of the JE situation over an extended duration in Yunnan Province. Inclusion of all cases reported in the province in the case-based surveillance system allowed for individual follow-up of all reported cases. The reporting of this study also followed the Strengthening the Reporting of Observational Studies in Epidemiology and the REporting of studies Conducted using Observational Routinely-collected health Data guidelines [22, 23].

Vaccine is the most effective and economic way in preventing infectious diseases [24–26]. Studies have shown that the routine use of JE vaccines results in dramatic decrease of cases and incidence rates [27–29]. Shift of cases to adults (as opposed to children) indicate that the older age groups are now at more risk of JE than the target population. To combat this, it is important to continue strengthening the vaccination program among children, as this will ensure that their risk of JE remains low even as they grow into adulthood. However, focus should also now be put to ensure that adults at risk in the endemic areas get vaccinated for JE. As it is, the vaccine is out-of-reach for many adults as they have to pay out of their pockets. The vaccine should therefore be made more affordable and available to the at-risk groups in the endemic areas. It should also be integrated into routine EPI for adults at risk. Whether repeat vaccination is needed during teenage-hood and/ or adulthood also need to be evaluated depending on efficacy and long-term protection provided by the vaccine.

Although most cases recorded in Yunnan Province were from locals, the number of cases from the bordering nations cannot be ignored. For total control and elimination of JE in Yunnan and even the whole nation, concerted efforts also need to be made by the neighboring countries. A multinational approach to JE surveillance and management should be strengthened. There is also need to now embark on contact investigation and management for every reported JE case according to the National JE Surveillance Guideline.

JE surveillance has been established or strengthened during the last few years in several countries. Since 2012, national surveillance programs have been established in Brunei, Democratic People’s Republic of Korea, and Timor Leste, and expanded in India and Nepal. However, the need to enhance the quality of JE surveillance is well recognized [17, 30]. Our study revealed the same situation - although the system has been in place for 13 years in Yunnan Province, there are aspects of its quality which still need to be improved. These include completeness of data (for example, vaccination history and treatment outcome) and its use for epidemiological investigations like contact tracing and follow up of cases.

Our study had several limitations: there were significant missing data in key variables like severity of disease, vaccination status, treatment outcome and testing of suspected cases, which introduce bias and limits analysis and interpretation; likewise, no significant rainfall, temperature or mosquito surveillance data – which are related to JE – were collected or analyzed. However, we plotted the trends in cases showing the months in which cases peaked. This may help in further analyzing the data to determine if weather and mosquito patterns play a role and point if monitoring the changes in climate and mosquito density and control, and predicting JE tendency using the mathematical model [31], could be useful.

## Conclusions

The 13-year JE surveillance data in Yunnan Province showed dramatic decrease of total incidence and the cases shift from children to adults. Improving the two-dose JE vaccination coverage, including access to adults at risk, and strengthening the JE surveillance system is needed to further control or eliminate JE in Yunnan Province.

## Supplementary information

**Additional file 1.** Multilingual abstracts in the five official working languages of the United Nations.

## Data Availability

Not applicable.

## Abbreviations

CDC: Centers for Disease Control and Prevention
CSF: Cerebrospinal fluid
EPI: Expanded Program on Immunization
JE: Japanese Encephalitis
JE1: 1st dose JE vaccine
JE2: 2nd dose JE vaccine
NNDRS: National Notifiable Disease Reporting System

## References

[1] Bharati K, Vrati S (2010). Japanese encephalitis vaccines: current status and future prospects. Proc Nat’l Acad Sci India - Section B: Biolog Sci.

[2] Liu Xinyu, Zhao Xin, Na Rui, Li Lili, Warkentin Eberhard, Witt Jennifer, Lu Xu, Yu Yongxin, Wei Yuquan, Peng Guohong, Li Yuhua, Wang Junzhi (2018). The structure differences of Japanese encephalitis virus SA14 and SA14-14-2 E proteins elucidate the virulence attenuation mechanism. Protein & Cell.

[3] World Health Organization. Japanese encephalitis. 2019. https://www.who.int/en/news-room/fact-sheets/detail/japanese-encephalitis. Accessed 9 May 2019.

[4] Campbell GL, Hills SL, Fischer M, Jacobson JA, Hoke CH, Hombach JM (2011). Estimated global incidence of Japanese encephalitis: a systematic review. Bull World Health Organ.

[5] Dan WU, Ning GJ, Yin ZD (2015). Epidemiological characteristics of Japanese encephalitis in China, 2011-2013. Chin J Vaccin Immun.

[6] Zhu QY, Hu XT, Kong Y, Zhang L, Ding ZR (2016). Seasonal distribution of Japanese encephalitis of Yunnan, 1952–2015. Modern Prev Med.

[7] Sohn YM (2000). Japanese encephalitis immunization in South Korea: past, present, and future. Emerg Infect Dis.

[8] Yen NT, Liu W, Hanh HD, Chang NY, Duong TN, Gibbons RV (2015). A model immunization programme to control Japanese encephalitis in Viet Nam. J Health Popul Nutr.

[9] Kumar Pant D, Tenzin T, Chand R, Kumar Sharma B, Raj BP (2017). Spatio-temporal epidemiology of Japanese encephalitis in Nepal, 2007-2015. PLoS One.

[10] Upreti SR, Lindsey NP, Bohara R, Choudhary GR, Shakya S, Gautam M (2017). Updated estimation of the impact of a Japanese encephalitis immunization program with live, attenuated SA 14-14-2 vaccine in Nepal. PLoS Negl Trop Dis.

[11] Lopez AL, Aldaba JG, Roque VG, Tandoc AO, Sy AK, Espino FE (2015). Epidemiology of Japanese encephalitis in the Philippines: a systematic review. PLoS Negl Trop Dis.

[12] Yearbook CS. China statistical Yearbook. 2018. http://www.stats.gov.cn/tjsj/ndsj/2018/indexeh.htm. Accessed 6 Dec 2018.

[13] Yunnan Statistical Yearbook. 2018. http://www.stats.yn.gov.cn/. Accessed 6 Dec 2018.

[14] Wang J, Zhang H, Sun X, Fu S, Wang H, Feng Y (2011). Distribution of mosquitoes and mosquito-borne arboviruses in Yunnan Province near the China-Myanmar-Laos border. Am J Trop Med Hyg.

[15] Bai L, Morton LC, Liu Q (2013). Climate change and mosquito-borne diseases in China: a review. Glob Health.

[16] World Health Organization SAGE Working Group on Japanese Encephalitis Vaccines (2014). Background paper on Japanese encephalitis vaccines—SAGE working group.

[17] Heffelfinger JD, Li X, Batmunkh N, Grabovac V, Diorditsa S, Liyanage JB (2017). Japanese encephalitis surveillance and immunization - Asia and Western Pacific regions, 2016. MMWR Morb Mortal Wkly Rep.

[18] Shui-gao JIN, JIANG T, Jia-qi MA (2006). Brief introduction of Chinese infection detection report information system. Chin Digit Med.

[19] Qiu XQ, Dong BQ, Yang JY, Lin M, Tan Y, Wu XH (2011). On-site assessment on the definition and classification of suspected cases in the manual of WHO Japanese Encephalitis Surveillance Standards. Zhonghua Liu Xing Bing Xue Za Zhi.

[20] Kakoti G, Dutta P, Ram Das B, Borah J, Mahanta J (2013). Clinical profile and outcome of Japanese encephalitis in children admitted with acute encephalitis syndrome. Biomed Res Int.

[21] Yin Z, Wang X, Li L, Li H, Zhang X, Li J, Ning G, Li F, Liang X, Gao L, Liang X, Li Y (2015). Neurological sequelae of hospitalized Japanese encephalitis cases in Gansu province, China. Am J Trop Med Hyg.

[22] Benchimol EI, Smeeth L, Guttmann A, Harron K, Moher D, Petersen I (2015). The REporting of studies conducted using observational routinely-collected health data (RECORD) statement. PLoS Med.

[23] Erik VE, Altman DG, Matthias E, Pocock SJ, Gøtzsche PC, Vandenbroucke JP (2008). Strengthening the reporting of observational studies in epidemiology (STROBE) statement: guidelines for reporting observational studies. BMJ..

[24] Pezzotti P, Bellino S, Prestinaci F, Iacchini S, Lucaroni F, Camoni L (2018). The impact of immunization programs on 10 vaccine preventable diseases in Italy: 1900-2015. Vaccine..

[25] Herndler-Brandstetter D, Grubeck-Loebenstein B (2007). The Efficacy of Vaccines to Prevent Infectious Diseases in the Elderly. Immunosenescence. Medical Intelligence Unit.

[26] Gao X, Li X, Li M, Fu S, Wang H, Zhi L (2014). Vaccine strategies for the control and prevention of Japanese encephalitis in mainland China, 1951–2011. PLoS Negl Trop Dis.

[27] Yang Y, Liang N, Tan Y, Xie Z (2016). Epidemiological trends and characteristics of Japanese encephalitis changed based on the vaccination program between 1960 and 2013 in Guangxi Zhuang autonomous region, southern China. Int J Infect Dis.

[28] Yao N, Wang Q, Zhou CB (2017). Epidemiological analysis of Japanese encephalitis before and after expanded program on immunization in Chongqing of China. Chin J Viral Dis.

[29] Deng SZ, Zhang HL, Liu XQ (2009). Analysis of epidemiological characteristics of Japanese encephalitis in Yunnan Province from 1976 to 2007. Endemic Dis Bull.

[30] World Health Organization (2014). Meeting report. Sixth biregional meeting on prevention and control of Japanese encephalitis.

[31] Panja P, Mondal SK, Chattopadhyay J (2018). Stability and bifurcation analysis of Japanese encephalitis model with/without effects of some control parameters. J Comp Appl Math.

