# Supplementary material for: Reduction patterns of Japanese encephalitis incidence following vaccine introduction into long-term expanded program on immunization in Yunnan Province, China
Source: Infect Dis Poverty. 2019 Dec 10;8:102. doi: 10.1186/s40249-019-0608-7 (PMC6902501; doi:10.1186/s40249-019-0608-7)
Supplement: Supplementary file 1 — Additional file 1. Multilingual abstracts in the five official working languages of the United Nations. [file 40249_2019_608_MOESM1_ESM.pdf]

Translation of the abstract into the five official working languages of the United Nations

أنماط الحد من الإصابة بالتهاب الدماغ الياباني بعد إدخال اللقاح في برنامج موسع طويل الأجل للتحصين في مقاطعة يونان في الصين

شياو تينغ هو، تشيونغ فين لي، تشاو ما ، زهي- زيان زهاو، لي-فانغ هي، تينغ- تينغ تانغ، وين يو، فيليب أويتي

ملخص

المعلومات الأساسية: التهاب الدماغ الياباني هو السبب الرئيسي لالتهاب الدماغ الفيروسي في مرحلة الطفولة على المستوى العالمي وفي الصين.يوصى بالتلقيح كاستراتيجية رئيسية لمكافحة التهاب الدماغ الياباني. في الصين، تم الإبلاغ عن معظم حالات التهاب الدماغ الياباني في المقاطعات الجنوبية الغربية، بما في ذلك مقاطعة يونان. في هذه الدراسة، قمنا بتحديد مقدار التحول الوبائي لمرض التهاب الدماغ الياباني في مقاطعة يونان من عام 2005 إلى عام 2017، والذي يغطي فترة ما قبل وما بعد إدخال لقاح التهاب الدماغ الياباني في البرنامج الموسع الروتيني للتحصين في عام 2007.

الطرق: استخدمنا البيانات التي تم جمعها بشكل روتيني في نظام مراقبة التهاب الدماغ الياباني القائم على الحالات من عام 2005 حتى عام 2017 في يونان. تم الإبلاغ عن حالات من المستشفيات ومراكز مكافحة الأمراض على مستوى المحافظة بما يتماشى مع المبدأ التوجيهي الوطني لمراقبة التهاب الدماغ الياباني. تم استخراج البيانات الوبائية وتحليلها وعرضها بالطرق المناسبة. تم تقدير التغطية بالتلقيح من جرعات التهاب الدماغ الياباني التي يتم إعطاؤها فعلياً والولادات الجديدة لكل عام.

النتائج: تم الإبلاغ عن إجمالي 4780 حالة التهاب دماغ ياباني (3077 تم تأكيدها مختبرياً، و 1266 سريرياً و437 مشتبه بها) في فترة الدراسة. ارتفع معدل الإصابة بالتهاب الدماغ الياباني (لكل 100,000 نسمة) من 0.95 عام 2005 إلى 1.69 عام 2007. مع زيادة التغطية بالتلقيح، انخفضت معدلات الإصابة بشكل مطرد من 1.16 في عام 2009 إلى 0.17 في عام 2017. ومع ذلك، ظلت الموسمية مماثلة على مر السنين، وبلغت ذروتها في يونيو - سبتمبر. سجلت معدلات الإصابة في بنا (المتاخمة لميانمار ولاوس) ودهونغ (المتاخمة لميانمار) وتشاوتونغ (وهي مقاطعة داخلية) أعلى معدلات الإصابة البالغة 2.3 و 1.9 و 1.6 على التوالي. كانت 97 ٪ من جميع الحالات بين السكان المحليين. مع زيادة التغطية بالتلقيح (وتناقص الإصابة)، انخفضت نسبة حالات التهاب الدماغ الياباني بين الأطفال الذين تقل أعمارهم عن 10 سنوات من 70 ٪ في عام 2005 إلى 32 ٪ في عام 2017 ، في حين زادت نسبة البالغين بين سن 20 سنة من 12 ٪ إلى 48 ٪. كان هناك عدد كبير من حالات التهاب الدماغ الياباني بنتائج علاج غير معروفة، خاصة في السنوات الأولى من نظام المراقبة.

الاستنتاجات: أظهرت بيانات مراقبة التهاب الدماغ الياباني التي استمرت 13 عاماً في مقاطعة يونان انخفاضاً كبيراً في معدل الإصابة الكلي وانتقالاً من الأطفال إلى البالغين. هناك حاجة لتحسين تغطية التلقيح، بما في ذلك الوصول إلى البالغين المعرضين للخطر، وتعزيز نظام مراقبة التهاب الدماغ الياباني لتحقيق مكافحة أكبر أو القضاء على المرض في المنطقة

Translated from English version into Arabic by Amal Imam, revised by Muhannad Albayk Jaam, through

TRANSLATORS WITHOUT BORDERS

云南省将乙脑疫苗纳入免疫规划前后乙脑流行病学特征

胡筱莛，李琼芬，马超，赵智娴，何丽芳，唐婷婷，余文，Philip Owiti

摘要

引言：乙脑是导致全球及中国儿童病毒性脑炎的主要病因之一。接种乙脑疫苗是控制乙脑的关键策略。在中国，包括云南在内的西南省份报告了大多数的乙脑病例。本研究分析了云南省 2005–2017 年将乙脑疫苗纳入免疫规划前后乙脑的流行病学特征。

方法：通过监测系统收集云南省 2005–2017 年乙脑监测数据，根据全国乙脑监测方案，病例报告来自医院和县级疾病预防控制中心，使用流行病学方法分析数据。乙脑疫苗接种率是根据每年乙脑疫苗实种数和出生儿童数进行估计。

结果：2005–2017 年云南省共报告 4780 例乙脑病例(实验室确诊病例 3077 例、临床确诊病例 1266 例、疑似病例

437 例)。乙脑报告发病率由 2005 年的 0.95/10 万上升至 2007 年的 1.69/10 万，随着疫苗接种率的提高，发病率从 2009 年的 1.16/10 万稳步下降到 2017 年的 0.17/10 万。乙脑发病有明显的季节性，每年在 6 月～9 月达到高峰。与缅甸、老挝接壤的版纳州、与缅甸接壤的德宏州及昭通市发病率较高，分别为 2.3/10 万、1.9/10 万及 1.6/10 万。97%的病例为本地病例。随着疫苗接种率的提高和发病率下降，10 岁以下儿童乙脑病例的比例从 2005 年的 70%下降到 2017 年的 32%，20 岁以上成人乙脑病例的比例从 12%上升到 48%。监测系统运转的早期，大量乙脑病例的转归记录不详。
结论：云南省 13 年乙脑监测数据显示，乙脑总体发病率显著下降，主要发病人群由儿童向成人转移。为进一步控制云南省乙脑发病，需要加强乙脑疫苗接种，尤其是高危地区的成人接种，进一步提高乙脑监测系统的监测质量。

Translated from English version into Chinese by Xiao-Ting Hu

Schémas de réduction de l'incidence de l'encéphalite japonaise après l'introduction d'un vaccin dans le programme élargi de vaccination à long terme dans la province du Yunnan, en Chine

Xiao-Ting Hu, Qiong-Fen Li, Chao Ma, Zhi-Xian Zhao, Li-Fang He, Ting-Ting Tang, Wen Yu, Philip Owiti

Résumé

Contexte: L'encéphalite japonaise (EJ) est l'une des principales causes d'encéphalite virale chez les enfants, tant au niveau mondial qu'en Chine. La vaccination, considérée comme une stratégie clé pour contrôler l'EJ est recommandée. En Chine, la plupart des cas d'EJ ont été rapportés dans les provinces du sud-ouest, incluant le Yunnan. Dans cette étude, nous quantifions la transition épidémiologique de l'EJ dans la province du Yunnan de 2005 à 2017, incluant les périodes avant et après l'introduction de la vaccination contre l'EJ dans le Programme élargi de vaccination (PEV) introduit en 2007.

Méthodes: Nous avons utilisé les données basées sur les cas de 2005 à 2017 dans le Yunnan et qui ont été collectées de manière régulière dans le système de surveillance de l'EJ. Des cas ont été signalés dans les centres de contrôle des maladies des hôpitaux et des comtés, conformément aux lignes directrices nationales sur la surveillance de l'EJ. Les données épidémiologiques ont été extraites, analysées et présentées de manière appropriée. La couverture vaccinale a été estimée à partir des doses réelles d'EJ administrées et des nouvelles naissances pour chaque année.

Résultats: Un total de 4 780 cas d'EJ (3 077 confirmés en laboratoire, 1 266 cas cliniques et 437 cas suspects) ont été rapportés durant l'étude. L'incidence de l'EJ (pour 100 000 habitants) est passée de 0,95 en 2005 à 1,69 en 2007. Avec l'augmentation de la couverture vaccinale, les taux d'incidence ont diminué progressivement, passant de 1,16 en 2009 à 0,17 en 2017. Cependant, la saisonnalité est restée similaire au fil des ans, atteignant son point le plus haut entre juin et septembre. Banna (à la frontière du Myanmar et du Laos), Dehong (à la frontière du Myanmar) et Zhaotong (une préfecture à l'intérieur du pays) affichaient les taux d'incidence les plus élevés de 2,3 ; 1,9 et 1,6, respectivement. De tous les cas, 97 % concernaient les résidents locaux. À mesure que la couverture vaccinale augmentait (et que l'incidence diminuait), la proportion de cas d'EJ chez les enfants de moins de 10 ans est passée de 70 % en 2005 à 32 % en 2017, tandis que chez les adultes de 20 ans et plus, elle est passée de 12 % à 48 %. Les résultats de traitement d'un grand nombre de cas d'EJ étaient inconnus, en particulier durant les premières années d'implantation du système de surveillance.

Conclusions: Les données de la surveillance de l'EJ durant 13 ans dans la province du Yunnan ont montré la diminution considérable de l'incidence totale de cas, ainsi qu'une différence entre le nombre de cas chez les enfants et les adultes. Il est nécessaire d'améliorer la couverture vaccinale, y compris pour les adultes à risque, et de renforcer le système de surveillance de l'EJ afin de mieux contrôler ou d'éliminer l'EJ dans la province.

Translated from English version into French by Caroline Girard, revised by DJUIKOUO Néhémie, through

Характер снижения заболеваемости японским энцефалитом вследствие введения вакцинации в расширенную долгосрочную программу иммунизации в провинции Юньнань Китая

Сяо-Тин Ху, Цюн-Фэнь Ли, Чао Ма, Чжи-Сянь Чжао, Ли-Фан Хэ, Тин-Тин Тан, Вэнь Юй, Филип Оуити

Резюме

Предпосылки: Японский энцефалит (ЯЭ) является основной причиной детского вирусного энцефалита как во всем мире, так и в Китае. В качестве основной стратегии контроля заболеваемости ЯЭ рекомендована вакцинация. В Китае большинство случаев заболеваемости ЯЭ было зарегистрировано в юго-западных провинциях, включая Юньнань. В настоящем исследовании нами была дана количественная оценка эпидемиологического сдвига ЯЭ в провинции Юньнань с 2005 по 2017 г., охватывающая время до и после введения вакцинации против ЯЭ в состав расширенной программы иммунизации в 2007 г.

Методы: Мы использовали регулярно собираемые данные за период с 2005 по 2017 г. из системы отслеживания отдельных случаев заболевания ЯЭ в провинции Юньнань. О случаях сообщали больницы ицентры по контролю заболеваемости уездного уровня в соответствии с Национальным руководством по контролю заболеваемости ЯЭ. Эпидемиологические данные были отобраны, проанализированы и представлены соответствующим образом. Охват иммунизацией оценивался по фактическим введенным дозам ЯЭ и новым рождениям за каждый год.

Результаты: Всего за период исследования сообщалось о 4780 случаях заболевания ЯЭ (3077 лабораторно подтвержденных, 1266 клинически подтвержденных и 437 предполагаемых). Заболеваемость ЯЭ (на 100 000 человек) выросла с 0,95 в 2005 г. до 1,69 в 2007 г. Вместе с ростом охвата вакцинацией заболеваемость постоянно снижалась с 1,16 в 2009 г. до 0,17 в 2017 г. Сезонность, однако, оставалась неизменной в течении этих лет, достигая пика в июне-сентябре. Наиболее высокая заболеваемость была в уездах Баньна (на границе с Мьянмой и Лаосом), Дэхун (на границе с Мьянмой) и Чжаотун (внутренний уезд), составившая 2,3, 1,9 и 1,6, соответственно. 97% всех случаев заболевания было среди местных жителей. По мере роста охвата вакцинацией (и снижения заболеваемости) число случаев заболеваемости ЯЭ среди детей младше 10 лет снизилось с 70% в 2005 г. до 32% в 2017 г., а среди населения старше 20 лет выросло с 12% до 48%. Было зарегистрировано большое число случаев заболевания ЯЭ с неизвестным исходом лечения, особенно в первые годы работы системы отслеживания.

Выводы: Данные 13-летнего отслеживания заболеваемости ЯЭ в провинции Юньнань показали резкое снижение заболеваемости и увеличение среднего возраста пациентов. Чтобы контролировать или искоренить ЯЭ в провинции необходимо улучшить охват вакцинацией, включая взрослых из групп риска, и повысить качество работы системы отслеживания заболеваемости ЯЭ.

Translated from English version into Russian by Alexander Somin, revised by Mikhail Kavalevich, through

Los patrones de reducción de la incidencia de encefalitis japonesa tras la introducción de vacunas en un programa ampliado de inmunización a largo plazo en la provincia de Yunnan, China

Xiao-Ting Hu, Qiong-Fen Li, Chao Ma, Zhi-Xian Zhao, Li-Fang He, Ting-Ting Tang, Wen Yu, Philip Owiti

Resumen

Antecedentes: la encefalitis japonesa (EJ) es una de las principales causas de encefalitis viral infantil, tanto a nivel mundial como en China. Se recomienda la vacunación como estrategia clave para controlar la EJ. En China, la mayoría de los casos de EJ se han notificado en provincias del suroeste, entre las que se incluye Yunnan. En este estudio, cuantificamos el cambio epidemiológico de la EJ en la provincia de Yunnan de 2005 a 2017, lo cual abarca el antes y después de la introducción de las vacunas contra la EJ en el Programa Ampliado de Inmunización de rutina (PAI) en 2007.

Métodos: utilizamos datos recopilados de forma rutinaria en el sistema de vigilancia de la EJ, el cual se basa en casos, desde 2005 hasta 2017 en Yunnan. Se notificaron casos de Centros para el Control de Enfermedades a nivel hospitalario y de condado de conformidad con la Guía Nacional de Vigilancia de la EJ. Los datos epidemiológicos se extrajeron, analizaron y presentaron de manera apropiada. La cobertura de la inmunización se estimó a partir de las dosis reales de EJ administradas y de los nuevos nacimientos para cada año.

Resultados: un total de 4780 casos de EJ (3077 confirmados en laboratorio, 1266 clínicos y 437 sospechosos) se reportaron en el período de estudio. La incidencia de la EJ (por 100.000 habitantes) aumentó de 0,95 en 2005 a 1,69 en 2007. Con el aumento de la cobertura de vacunación, las tasas de incidencia disminuyeron constantemente de 1,16 en 2009 a 0,17 en 2017. Sin embargo, la estacionalidad se mantuvo similar a lo largo de los años, alcanzando su punto máximo en junio-septiembre. Banna (fronteriza con Myanmar y Laos), Dehong (fronteriza con Myanmar) y Zhaotong (una prefectura interior) tuvieron las tasas de incidencia más altas de 2,3, 1,9 y 1,6, respectivamente. El 97 % de todos los casos fue entre residentes locales. A medida que la cobertura de vacunación aumentó (y la incidencia disminuyó), la proporción de casos de EJ entre niños menores de 10 años disminuyó del 70 % en 2005 al 32 % en 2017, mientras que la de adultos mayores de 20 años aumentó del 12 % al 48 %. Hubo una gran cantidad de casos de EJ con resultados de tratamiento desconocidos, especialmente en los primeros años del sistema de vigilancia.

Conclusiones: los datos de vigilancia de la EJ de 13 años en la provincia de Yunnan mostraron una disminución drástica de la incidencia total y un cambio de niños a adultos. Es necesario mejorar la cobertura de vacunación, incluido el acceso de los adultos en riesgo, y reforzar el sistema de vigilancia de la JE para controlar mejor o eliminar la EJ en la provincia.

Translated from English version into Spanish by Maria Luz Puerta, revised by Mayra Léon, through

TRANSLATORS WITHOUT BORDERS
